# Diversity of Methane-Oxidizing Bacteria in Soils from “Hot Lands of Medolla” (Italy) Featured by Anomalous High-Temperatures and Biogenic CO_2_ Emission

**DOI:** 10.1264/jsme2.ME16087

**Published:** 2016-09-17

**Authors:** Martina Cappelletti, Daniele Ghezzi, Davide Zannoni, Bruno Capaccioni, Stefano Fedi

**Affiliations:** 1 Department of Pharmacy and Biotechnology, University of BolognaVia Irnerio 42, 40126, BolognaItaly; 2 Department of Biological, Geological and Environmental Sciences, University of BolognaPiazza di Porta S. Donato 1, 40126, BolognaItaly

**Keywords:** methanotrophs, methane-oxidizing bacteria, *pmoA* gene, Terre Calde di Medolla (“Hot Lands of Medolla”), soil high temperature

## Abstract

“Terre Calde di Medolla” (TCM) (literally, “Hot Lands of Medolla”) refers to a farming area in Italy with anomalously high temperatures and diffuse emissions of biogenic CO_2_, which has been linked to CH_4_ oxidation processes from a depth of 0.7 m to the surface. We herein assessed the composition of the total bacterial community and diversity of methane-oxidizing bacteria (MOB) in soil samples collected at a depth at which the peak temperature was detected (0.6 m). Cultivation-independent methods were used, such as: i) a clone library analysis of the 16S rRNA gene and *pmoA* (coding for the α-subunit of the particulate methane monooxygenase) gene, and ii) Terminal Restriction Fragment Length Polymorphism (T-RFLP) fingerprinting. The 16S rRNA gene analysis assessed the predominance of *Actinobacteria*, *Acidobacteria*, *Proteobacteria*, and *Bacillus* in TCM samples collected at a depth of 0.6 m along with the presence of methanotrophs (*Methylocaldum* and *Methylobacter*) and methylotrophs (*Methylobacillus*). The phylogenetic analysis of *pmoA* sequences showed the presence of MOB affiliated with *Methylomonas*, *Methylocystis*, *Methylococcus*, and *Methylocaldum* in addition to as yet uncultivated and uncharacterized methanotrophs. Jaccard’s analysis of T-RFLP profiles at different ground depths revealed a similar MOB composition in soil samples at depths of 0.6 m and 0.7 m, while this similarity was weaker between these samples and those taken at a depth of 2.5 m, in which the genus *Methylocaldum* was absent. These results correlate the anomalously high temperatures of the farming area of “Terre Calde di Medolla” with the presence of microbial methane-oxidizing bacteria.

Methane (CH_4_) is the most abundant organic gas in the atmosphere; it represents the second most important green-house after carbon dioxide (CO_2_) (11, 35) and is strictly involved in carbon cycle processes. Therefore, processes with the ability to consume atmospheric CH_4_ may play important roles in preventing climate change (21, 28). The origin of CH_4_ in the atmosphere is related to different anthropogenic (*e.g.*, rice paddies, livestock farms, biomass burning, oil and gas mining, and waste disposal) and natural (*e.g.*, wetlands, termites, oceans, freshwaters, and seepage from deep reservoirs in both sedimentary and volcanic environments) sources (6, 37). The total amount of atmospheric CH_4_ is 520 Tg year^−1^, 90% of which is oxidized by photochemical reactions in the troposphere while 10% is removed by microbiological activities (15).

Methanotrophs (also named Methane-Oxidizing Bacteria, MOB) are responsible for most of the biological processes of CH_4_ oxidation. MOB are a unique group of Gram-negative aerobic bacteria that metabolize CH_4_ as the only source of carbon and energy (15). They are ubiquitous microorganisms in nature that are able to adapt to different environmental conditions (10). Methane consumption by MOB occurs through an oxidation reaction led by the methane monooxygenase enzyme (MMO), which catalyzes the conversion of methane in methanol (26). This is the first step in the metabolic pathway of methanotrophs, which ends with the production of CO_2_ (24). Methanotrophs have been classified into two distinct taxonomic groups based on phenotypic and genotypic properties. Type I methanotrophs belong to the γ-subdivision of *Proteobacteria*, whereas type II methanotrophs belong to the α-subdivision of *Proteobacteria* (15). Two types of MMO systems have been identified: a soluble cytoplasmic complex (sMMO) and membrane-bound particulate system (pMMO) (18). pMMO genes are universal in MOB, with the possible exception of *Methylocella palustris* (11). In contrast, sMMO genes are restricted to type II methanotrophs with the exception of certain representatives of two type I genera (27).

The application of molecular biological tools has greatly facilitated the study of methanotroph communities in natural environments. The *pmoA* gene, which encodes the α-subunit of pMMO, has been widely used as a phylogenetic marker for the identification of methanotrophs through cultivationin-dependent approaches. The *pmoA* sequence has provided information on the diversity of these organisms in different environments (19, 25).

“Terre Calde di Medolla” (TCM) (literally, “Hot Lands of Medolla”) is an ancient toponym that dates back to 1893 (6, 33) and describes a heating surface phenomenon affecting agricultural soils located in the area of Medolla, a small town in the province of Modena (Italy) (Fig. 1A). This phenomenon has a patchy distribution, with a sub-circular shaped area (a few meters in diameter) in which temperatures up to ≅50°C were measured (Fig. 1C and B). The anomalously warm areas are easily recognizable during winter because snow melts within them and during summer when soil heating leads to the death of corn plants (6). Previous studies conducted in 2012 reported a clear correlation between soil heating and gas seepage (CH_4_ and CO_2_) (29). As shown in Fig. 2B, the maximum temperature (42.2°C) is reached at a depth of 0.6 m, where horizons with high permeability are present (loamy sandy layer and sandy layer). The CO_2_/CH_4_ flux ratio (φCO_2_/ φCH_4_) increases from this level to the surface by three orders of magnitude, from values <1 to >1,000 (Fig. 2D). The chemical composition of soil gases together with the gradual increase of ^12^C in CO_2_ and decrease in CH_4_ at shallower depths (from 0.7 m to 0.25 m, Fig. 2A) indicate that the marked increase in the CO_2_/CH_4_ flux ratio is due to the biological oxidation of rising CH_4_, suggesting the occurrence of biological methane oxidation processes in the most aerated layers. Conceptual modeling and numerical simulations have indicated that the exothermic nature of the CH_4_→CO_2_ conversion (800 KJ mol^−1^ of CH_4_) represents a heat source with the ability to produce the soil thermal anomalies observed in TCM soil (29). This previous finding and thermodynamic prediction prompted us to verify the hypothesized correlation between methanotrophs and soil hot temperatures by examining microbial methanotrophic diversity in TMC soil samples using a cultivation-independent approach. Three soil samples were collected at different ground depths (0.6, 0.7, and 2.5 m). A clone library analysis of the 16S rRNA gene and *pmoA* gene was performed on 0.6-TCM soil in order to characterize the total bacterial community and define the MOB fraction, respectively. The methanotrophic community composition identified in 0.6-TCM soil was compared to those detected in 0.7- and 2.5-TCM soil by Terminal Restriction Fragment Length Polymorphism (T-RFLP) fingerprinting of the *pmoA* gene. The results presented here strongly support earlier geophysical models implicating the presence of microbial methane-oxidizing activity in the farming area of “Terre Calde di Medolla” in the anomalous heating of soil.

## Materials and Methods

### Sample collection

During a field campaign conducted in July 2013, a 2.5-m-deep piezometer was drilled within an area selected on the basis of the presence of an anomalously high surface soil temperature (36.4°C) and significant CO_2_ fluxes (up to 103.4 g m^−2^ d^−1^) (Fig. 2B and D). During drilling, three different soil samples were collected within the sediment corer at depths of 0.6, 0.7, and 2.5 m (hereafter referred to as 0.6-TCM, 0.7-TCM, and 2.5-TCM) and stored within small polystyrene tubes kept at −20°C for later use. During drilling, CO_2_ and CH_4_ fluxes were also measured at 0.1-m intervals to a depth of 0.80 m and then at intervals of 0.5 m thereafter.

### Extraction of DNA

Genomic DNA was extracted from 0.35 g of 0.6-TCM, 0.7-TCM, and 2.5-TCM soils in duplicate using the Power Soil DNA isolation kit (MOBIO Laboratories, Solana Beach, CA, USA) according to the manufacturer’s instructions with some modifications. Briefly, 0.35 g of each soil sample was incubated in IRS solution supplied with 5 mg mL^−1^ proteinase K and 8 mg mL^−1^ lysozyme at 37°C for 30 min. After the addition of 0.1% of SDS, cells were further disrupted by bead beating for three cycles of 1 min each. DNA was eluted in a final volume of 50 μL and stored at −20°C in milli-Q water.

### Construction of gene clone libraries and RFLP screening

Isolated genomic DNA was used for PCR amplification of the 16S rRNA gene and/or functional genes (*pmoA* and *mmoX*) using the primers listed in Table 1. Bacterial DNA (1 μL of 10× diluted genomic DNA) was added to a 50-μL (final volume) mixture containing 1.25% (v/v) DMSO and 1.25 U Taq polymerase (Thermo Scientific). The following conditions were used to amplify the 16S rRNA gene: at 94°C for 4 min for initial denaturation, 30 cycles (at 94°C for 40 s and 55°C for 40 s for alignment and at 72°C for 40 s for elongation), and at 72°C for 15 min for the final extension step. The *pmoA* gene was amplified using the following PCR program: at 94°C for 4 min, 30 cycles (at 94°C for 1 min, 56°C for 1 min, and 72°C for 1 min), and a final elongation at 72°C for 20 min. The PCR reactions and cycling conditions used for the amplification of *mmoX* were as described for *pmoA*, except for an annealing temperature of 60°C.

PCR amplification products were confirmed by electrophoresis with a 1% (w/v) agarose gel, purified with the Qiagen PCR purification kit (Qiagen, Hilden, Germany). PCR products were ligated into the pCRII vector supplied with the TOPO TA cloning kit (Invitrogen, San Diego, CA, USA), according to the manufacturer’s instructions, and cloned into *Escherichia coli* DH5α for clone library construction.

A Restriction Fragment Length Polymorphism (RFLP) analysis was performed on at least 100 clones from each library. Individual colonies containing inserts of the appropriate size were suspended in 20 μL of TE pH 8 and boiled for 5 min. Cell debris was removed by centrifugation and 1-μL portions of the supernatant were used as templates in PCR mixtures to re-amplify the gene inserts that were further used in restriction digestion with tetrameric restriction enzymes. The genes were digested with 5 U of each enzyme at 37°C for 3 h. Restriction profiles were analyzed by 2% agarose gel electrophoresis with high-resolution agarose (Metaphor, Tebu-bio). Clones were grouped manually based on restriction patterns.

Regarding sequence identification, plasmids (for the 16S rRNA gene) or PCR products (for *pmoA*) were purified from one representative clone of each group with a Qiagen plasmid purification kit or QIAquick PCR Purification kit (Qiagen). Sequencing was performed by the BMR Genomics Service (Padova, Italy) using T7 and T3 primers (Table 1). Sequences were checked for chimeras using the CHECK_CHIMERA program at the Ribosomal Database Project (RDP) (https://rdp.cme.msu.edu/).

### Sequence analysis

An analysis of the 16S rRNA gene and *pmoA* gene sequences obtained from the libraries was performed using the Ribosomal Database Project (8) as well as BLASTn (1) hits against GenBank to generate the best hits. A phylogenetic tree of the derived *pmoA* gene nucleotide sequences was created using Geneious Tree Builder with the Juke-Cantor genetic distance model and using the neighbor-joining method. One thousand parametric bootstrap replications were simultaneously computed to statistically consolidate the branching topology of the inferred tree.

### T-RFLP analysis

A T-RFLP analysis was performed for each sample in triplicate as described previously (7). The same PCR primers and conditions as those described above were used, although the forward primer A189f was labeled at the 5′ end with hexachlorofluorescein dye (Hex).

After purification with Qiaquick spin columns (Qiagen), approximately 200 ng of fluorescently labeled PCR amplification products were digested with 10 U of the restriction enzymes (Roche) *Msp*I and *Hae*III. Digestion was performed in a total volume of 20 μL at 37°C for 3 h. The digests obtained were sent to BMR Padova for automated DNA sequencing.

### T-RF profile and statistical analyses

The lengths of the labeled fragments were assessed by comparison with an internal standard (ROX-labeled GS500) using Peak Scanner version 1.0 software (Applied Biosystems). A tolerance limit of +/− 2 bp was used for peak assignment on the sizing accuracy of T-RFs ranging from 35/50 to 500 bp. In order to avoid the detection of primers and uncertainties of size determination, terminal fragments smaller than 50 bp were typically excluded from the analysis. In the digestion of *Hae*III only, T-RFs ranging from 35 to 50 bp were taken in the analysis because the *Hae*III restriction sites included in this size range were expected in *pmoA* amplicons. A 1% threshold was used to define the baseline. A peak height threshold of 50 fluorescence units was used in the initial analysis of the electropherogram. In order to compare the T-RFLP data obtained from the different soil samples being analyzed, T-RFLP profiles were normalized as previously described by Stralis-Pavese *et al.* (36).

T-RF peaks were regarded as binary characters and analyzed using NTSYS software as described previously (13) to calculate the distance between soil samples analyzed in terms of T-RFs detected.

### Nucleotide sequence accession numbers

The *pmoA* gene sequences identified in this study have been deposited in the DDBJ/EMBL/GenBank databases under accession numbers from KX035110 to KX035129.

## Results

### Microbial community characterization of soils sampled at a depth of 60 cm by clone library screening

#### 16S rRNA gene sequences

A total of 100 randomly selected non-chimeric rDNA clones containing inserts from the 16S rRNA gene clone library were subjected to RFLP analyses (with the tetrameric restriction enzymes *Hae*III, *Alu*I, and *Rsa*I) and placed into groups based on their RFLP patterns. One representative clone for each group was sequenced. A total of 22 bacterial phylotypes were found to be affiliated with 7 distinct phyla through a RDP analysis (Table 2).

Most clones in the clone library and T-RFLP analyses belonged to the phyla *Proteobacteria* (25%) and *Actinobacteria* (24%) followed by *Acidobacteria* (17%) and the genus *Bacillus* in the phylum *Firmicutes* (16%) (Table 2). 16S rRNA gene phylotypes associated with the oxidation of CH_4_ and methanol represented 12% of all clones screened from the library and were all detected within the phylum *Proteobacteria*. The 16S rRNA gene sequences of two methanotrophs belonged to the genera *Methylobacter* and *Methylocaldum*, both of the *Gammaproteobacteria* class, while one methylotroph belonged to the genus *Methylobacillum* of the *Betaproteobacteria* class.

#### pmoA sequences

A molecular analysis of the *pmoA* gene was used to characterize the methane-oxidizing microbial community, thereby identifying the most representative strains involved in CH_4_ consumption.

The genes coding for the α-subunit of both types of methane monooxygenases (particulate and soluble MMO) were targeted in the present study. The *pmoA* gene encoding the α subunit of the pMMO was successfully amplified with the primer sets, A189f/mb661r and A189f/A682r, while no PCR product was obtained using the primers for the α subunit of the soluble isoform of the methane monooxygenase (mmoXA/mmoXD and mmoX206f/mmoX866r, Table 1) (data not shown). Corresponding to each primer set (amplicon lengths of 510 bp and 525 bp with A189f/mb661r and for A189f/A682r, respectively), two clone libraries were constructed. A total of 200 clones were screened from the two clone libraries through the RFLP analysis using *Hae*III and *Msp*I restriction enzymes. Similar to 16S rRNA gene screening, the representative clone of each RFLP group was detected and sequenced in both strands. The *pmoA* sequences were aligned, analyzed through a BLAST search and grouped (group A–H), if possible, using a threshold of 90% sequence identity (Table S1). Sequences that did not show any affiliation with *pmoA* sequences were excluded from further analyses.

A distance-based neighbor-joining tree was constructed with the *pmoA* sequences obtained from the two libraries and their closely related reference sequences obtained from the GenBank database (Fig. 3, Table 3). The phylogenetic analysis of the *pmoA* sequences revealed the presence of MOB in soil related to the genera *Methylocystis* (group A), *Methylococcus* (groups F, D, and E), *Methylocaldum* (group G), and *Methylomonas* (group H) (Fig. 3), indicating wide biodiversity in terms of bacteria associated with CH_4_ consumption. Moreover, two groups of clones (groups B and C in Fig. 3 and Table 3) clustered with the *pmoA* clones of uncultured or uncharacterized methanotrophs without a defined taxonomic affiliation (uncultured *pmoA* groups I and II, Fig. 3). In the clone library with A189f/A682r, one clone corresponded to a partial *amoA* gene that clustered in the phylogenetic analysis with the *amoA* gene of *Nitrosospira* sp. (Fig. 3, Table 3). The clone groups C and D, belonging to uncultured methanotrophs and the genus *Methylococcus*, respectively, were the most dominant in both libraries; however, the percentage of clones belonging to each clone group was different (Table 3). In the A189f/mb661r library, 49% and 18% of clones belonged to groups C and D, respectively, while in the A189f/A682r library, 57% and 23% of clones belonged to groups D and C, respectively. The clones of group B represented 10% of all the clones characterized in the A189f/ A682r library, while the clones of group A as well as those of groups F, G, and H were detected in the A189f/mb661r library only. The latter represented 1%, 17%, and 2%, respectively, of all clones characterized in the A189f/mb661r library. Notably, group G was the third most represented phylotype in the library and was closely related to the genus *Methylocaldum*. The different results obtained from the screening of the two libraries may be related to the different abilities of the two primer sets (A189f/A682r and A189f/ mb661r) to assess methanotroph diversity in soil (4). In the present study, the A189f/mb661r primer set retrieved the largest diversity of methanotroph *pmoA* sequences. Therefore, this primer set was used in T-RFLP analyses. As shown in Table 3 and Fig. 4 (0.6-m *Hae*III and 0.6-m *Msp*I), the *pmoA-*based T-RFLP profiles obtained from 0.6-TCM soil correlated with the results of clone library screening. Additionally, the minor T-RFs that were not affiliated with any *pmoA* sequence from the libraries may represent new methanotrophic species. Among these, the T-RFs not corresponding to any expected digestion fragments with a relative area greater than 1% were 65, 78, 93, 144, and 210 bp in the *Hae*III digestion, and 63 and 75 bp in the *Msp*I digestion (0.6-m *Hae*III and 0.6-m *Msp*I in Fig. 4).

### T-RFLP analysis to compare MOB at different ground depths (soils sampled at 0.6, 0.7, and 2.5 m)

A T-RFLP analysis of the amplified *pmoA* gene was used to elucidate differences and similarities between sediments sampled at different ground depths (0.6, 0.7, and 2.5 m) in TCM.

On the basis of the presence or absence of terminal restriction fragments (T-RFs) in the profiles shown in Fig. 4, an analysis of Jaccard’s distance (JD) was performed in order to compare the MOB communities present in soil collected at different depths (Table S1). A similarity index of 1.0 indicated that communities have strong similarities. Jaccard’s index (S_j_) between 0.6- and 0.7-TCM soil was 0.905, demonstrating stability in the microbial composition of the two soil samples. A lower degree of similarity in the MOB community composition was revealed between 0.6-TCM/0.7-TCM soil and 2.5-TCM soil (JD of 0.6–0.7) (Table S1).

**Fig. 1:**
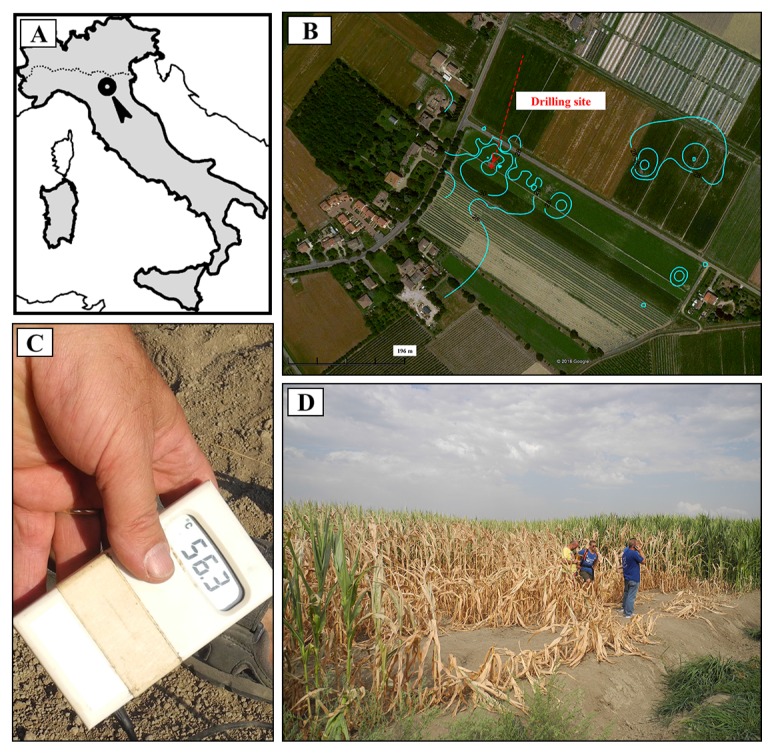
A) A map of Italy showing the location of Terre Calde di Medolla (“Hot Lands of Medolla”); B) Aerial photo showing the temperature distribution at Terre Calde di Medolla reported as isothermal curves. The location of the sampling site with soil temperatures of 42°C is also indicated; C) Extreme values of soil temperature ≅55°C measured within the area of Terre Calde di Medolla a few months after the seismic sequence that occurred in May–June 2012 in the Emilia region; D) Picture of a thermally anomalous area with clear signs of affected vegetation.

**Fig. 2:**
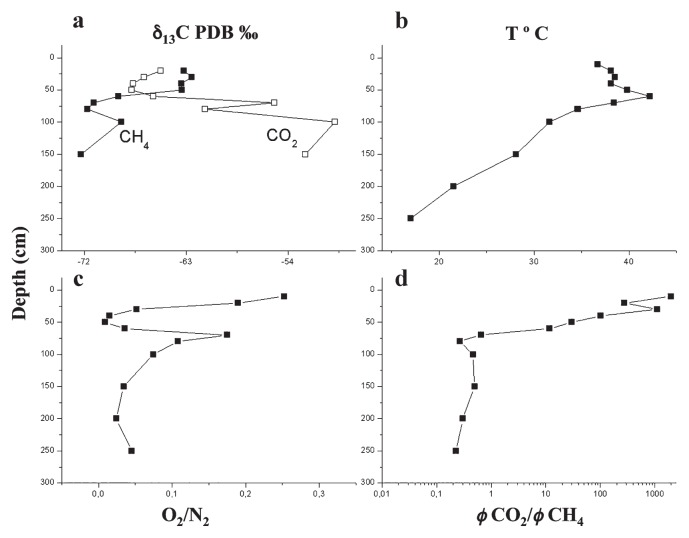
Vertical profiles of temperature and chemical and isotopic compositions of soil gases collected from the surface downwards to a depth of 2.5 m [modified from Capaccioni *et al.* (6)]. A) Vertical profile of the isotopic composition of CH_4_ and CO_2_; ^13^C/^12^C-CO_2_ and ^13^C/^12^C-CH_4_ values are expressed as δ_13_C-CO_2_ VPDB (Vienna Pee Dee Belemnite) ‰ and δ_13_C-CO_2_ PDB ‰, respectively. B) Vertical profile of temperatures measured with depth using a digital thermometer, C) and D) Vertical profiles of O_2_/N_2_ and CO_2_/CH_4_ flux ratios measured during piezometer drilling.

T-RFs corresponding to the clone group G (99 bp T-RF with *Hae*III, 76 bp T-RF with *Msp*I) were present in the T-RFLP profiles generated from 0.6-TCM and 0.7-TCM soil, while their abundance was below the detection limit in 2.5-TCM soil. The presence of the group G *pmoA* clone in 0.6-TCM and 0.7-TCM soil that correlated to the genus *Methylocaldum* may be associated with the higher temperatures at these ground depths (Fig. 1 and 5). Further differences were observed in the presence of unaffiliated T-RFs. The 2.5-m T-RFLP analysis did not show *Hae*III T-RFs 78 and 93 bp or *Msp*I T-RF 76 bp, which were present in the 0.6-TCM and 0.7-TCM profiles. The differences between 0.6-TCM and 0.7-TCM included *Hae*III T-RF 210 bp and T-RFs associated with the secondary restriction sites of the *pmoA* sequences (*Msp*I T-RF 373 and *Hae*III T-RF 351).

## Discussion

The area known as TCM in the Po river valley (Italy) has been attracting interest since 1893 (6, 29, 33) due to its unusual ground temperatures that reach up to 50°C at a depth of 0.6 m, which exceed local average values. This phenomenon is associated with diffuse CH_4_ and biogenic CO_2_ seepage (6), and conceptual modeling linked to numerical simulations have suggested that the exothermic nature of the CH_4_→CO_2_ conversion (800 KJ mol^−1^ of CH_4_) represents a heat source with the ability to produce the thermal anomalies observed in TCM soil (29). Indirect support for this hypothesis is provided by an early study on permafrost, which quantified the specific heat released by methanotrophy to be in the order of 50 MJ (kg_CH4_)^−1^ (23).

The 16S rRNA gene clone library analysis of 0.6-TCM soil (this work) indicates the predominance of bacteria belonging to the phyla *Proteobacteria* (with representatives of the three classes *Alpha*-, *Beta*-, and *Gammaproteobacteria*), *Actinobacteria*, and *Acidobacteria* (17). Among *Proteobacteria*, two methanotrophic genera belonged to *Gammaproteobacteria*, while one methylotrophic genus in the library belonged to *Betaproteobacteria*. A total of 12% of the clones of the 16S rRNA gene library, constructed with the universal 27f/1492r primer set, are representative of methanotrophs and/or methylotrophs, suggesting the occurrence of methane-oxidizing activities in high-temperature soil, *i.e.* 0.6-TCM soil.

An examination of the *pmoA* clone libraries of 0.6-TCM soil revealed the large biodiversity of methanotrophs including various MOB affiliated to *Methylomonas*, *Methylococcus*, *Methylocystis*, and *Methylocaldum*. In the A189f/mb661r *pmoA* library, most clones (≅50%) were found within group C, which represents uncultivated methanotrophic bacteria related to methanotrophs isolated from upland grassland soil consuming atmospheric CH_4_ and a saline alkaline environment (21, 34). In the A189f/A682r library of 0.6-TCM soil, most clones (57%) were found within group D, which shared a maximum of 90% nucleotide similarity with the uncultivated methanotrophic bacteria identified in paddy fields cultivated with rice over a long period of time (16). The relative abundance of the two groups in the two clone libraries may be associated with the different specificities of the two primer sets used to amplify the molecular marker gene *pmoA*. In particular, the primer pair (A189f/mb661r) was able to detect a larger set of *pmoA* sequences and did not amplify any *amoA* from the 0.6-TCM extract. This is in line with previous findings showing the superior ability of A189f/mb661r over A189f/A689r to detect a larger part of methanotrophic bacteria and its higher specificity to amplify *pmoA* (4, 9, 31). Therefore, this primer set was used in the T-RFLP analysis of *pmoA* in 0.6-TCM soil. T-RFs corresponding to all the groups detected in the clone libraries were found in T-RFLP fingerprinting. Although the genus *Methylobacter* was detected in the 16S rRNA gene clone library, but not in the *pmoA* clone library, *Msp*I and *Hae*III T-RFs (505 and 350 bp, respectively) were found in T-RFLPs, which may correspond to *Methylobacter pmoA* digestion fragments on the basis of the *Msp*I T-RFLP analysis reported by Horz *et al.* (20) and also the *in silico Hae*III digestion of representatives of this gene present in the database (*e.g.* the *pmoA* gene with GenBank ID EU124862 and JQ038155).

**Fig. 3:**
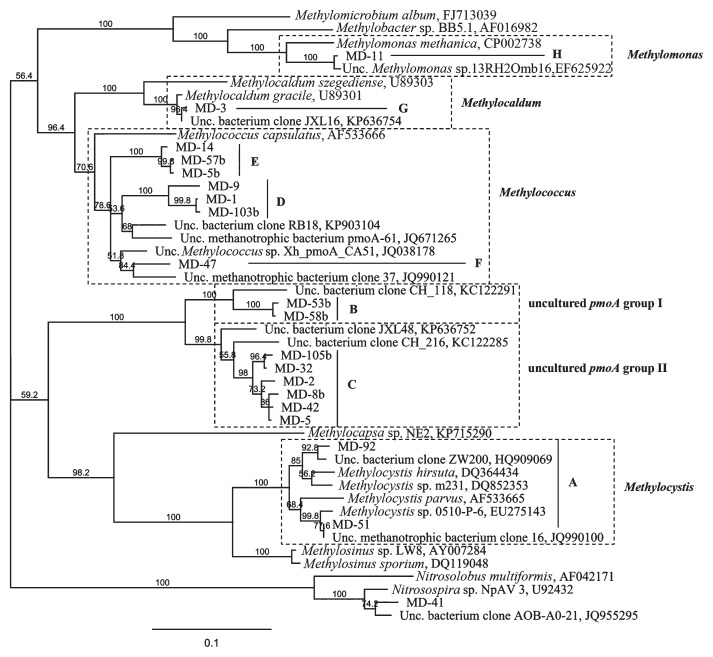
Phylogenetic tree of partial *pmoA* and *amoA* nucleotide sequences retrieved from an analysis of A189f/A682r and A189f/mb661r clone libraries from 0.6-TCM soil. *pmoA* clones are grouped into eight groups (A–H) based on at least 90% nucleotide sequence identity. GenBank accession numbers are shown for sequences of cultured methanotrophs and clones from other studies. The bar indicates 10% sequence divergence. Bootstrap values are given and based on 500 data resampling. Boxes marked by dashed lines show the phylogenetic affiliations of the *pmoA* clones from 0.6-TCM soil.

**Fig. 4:**
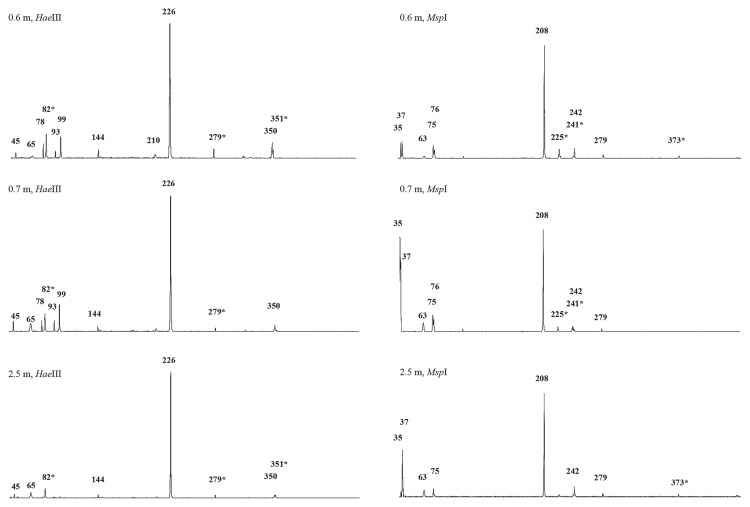
Comparison of representative T-RFLP patterns of *pmoA* products amplified from DNA extracted from different ground depths of TCM (0.6-, 0.7- and 2.5-TCM soil). The lengths of significant T-RFs (signal intensity greater than the threshold of 1%) are reported. See Table 3 for the assignment of T-RFs to methanotrophic species/clones revealed from clone library screening. Putative peaks representing partial digestion are depicted by asterisks.

**Fig. 5:**
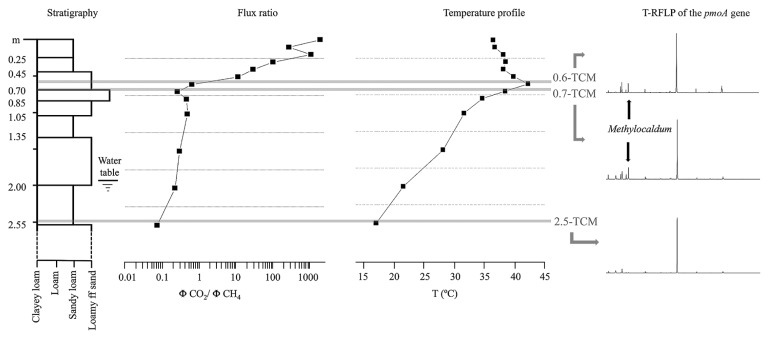
Visual representation of the vertical variability of sedimentological features (from the surface downwards to a depth of 2.5 m) associated with the corresponding profiles of temperature, the CO_2_/CH_4_ flux ratio, and T-RFLP patterns (*Hae*III-based digestion) representing methanotrophic diversity in soil collected at depths of 0.6, 0.7, and 2.5 m. The T-RF corresponding to the genus *Methylocaldum* is highlighted in samples from depths of 0.6 and 0.7 m.

It is important to note that the T-RFLP analysis of the *pmoA* gene demonstrated that the vertical distribution of MOB (at depths of 0.6, 0.7, and 2.5 m) is relatively stable in TMC soil, independently of differences in oxygen availability, as has been reported in studies on sediments from Lake Constance (CH) (30, 32). In TCM soil, CO_2_ production linked to CH_4_ consumption was detected at a depth of 0.7 m to the surface, indicating that methanotrophic activity occurs at shallow depths at which O_2_ is present, whereas MOB activity is absent at a depth of 2.5 m at which CH_4_ is present and O_2_ is absent. The main difference observed with depth was the presence of MOB belonging to group G (Fig. 3, 4 and 5). T-RFLP comparisons highlighted the presence of T-RFs (in the *Hae*III- and *Msp*I-based profiles) corresponding to *Methylocaldum*-affiliated bacteria in soil collected at depths of 0.6 and 0.7 m. These T-RFs were below the detectable limit in 2.5-TCM soil (Fig. 4 and 5). In 0.6-TCM soil, the presence of the genus *Methylocaldum* was detected by analyzing both the gene markers, the *pmoA* and 16S rRNA genes. In particular, the *pmoA* sequence of the clone group G was phylogenetically correlated with *Methylocaldum gracile* (nt identity 99%). Bacteria belonging to the genus *Methylocaldum* are widely distributed in nature (3). Their habitats are thermal springs, activated sludge, arable soils, silage waste, and manure (14, 38). *Methylocaldum gracile* grows at 20°C and *Methylocaldum tepidum* at 30°C, with both growing optimally at 42°C and at a maximal temperature of 47°C (5). The presence of detectable *Methylocaldum*-related T-RFs at ground depths of 0.6 and 0.7 m may be related to an optimal growth temperature (42°C) present in this soil layer along with advantageous oxygen concentrations and other undefined favorable environmental conditions occurring at depths of 0.7 and 0.6 m, but not at 2.5 m. Additional T-RFs, not related to any clones in the *pmoA* library, distinguished 0.6-TCM soil from 2.5-TCM soil; however, the contribution of these putative MOB species has not yet been elucidated. The optimal conditions for the CH_4_ conversion are present at depths of approximately about 0.7–0.6 m, at which a significant increase in temperature was detected and bacteria affiliated with the genus *Methylocaldum* were identified. Chemical and isotopic data together with the presence of MOB appear to indicate that this process may even occur at depths >0.6 m. However, at deeper levels, the lack of free oxygen does not appear to allow efficient and complete CH_4_→CO_2_ conversion. This is also spatially associated to the absence of significant warming phenomena and not detectability of *Methylocaldum*-affiliated bacteria.

In conclusion, the results of the present study support and emphasize previous findings by Capaccioni *et al.* (6) and Nespoli *et al.* (29) in which strong biological methane-oxidizing activity in TMC soil was proposed to be related to the anomalous temperatures detected at a depth of 0.6 m. Accordingly, the spatial associations between the anomalously high temperatures in TCM soil, the consumption of CH_4_ and O_2_, the production of biogenic CO_2_ at shallow levels, and the detection of MOB all agree with the occurrence of microbial CH_4_→CO_2_ conversion and soil heating. Although the phenomenon described here occurs in a local farming area in Italy, the importance of our work lies in the relationship we found between methanotrophy and soil heating, a topic that deserves public interest for its implications in climate warming and methane release.

## Supplementary Information



## Figures and Tables

**Table 1:** Primers used in the present study for PCR amplification and sequencing.

Target gene or scope	Primer set	Sequence (5′ to 3′)	Fragment length	Reference
16S rRNA gene	27f 1492r	AGAGTTTGATCHTGGCTCAG	1465 bp	(12)
		TACGGYTACCTTGTTACGACTT		
*pmoA*	A189f A682r	GGNGACTGGGACTTCTGG	525 bp	(18)
		GAASGCNGAGAAGAASGC		
	A189f mb661r	GGNGACTGGGACTTCTGG	510 bp	(9)
		CCGGMGCAACGTCYTTACC		
*mmoX*	mmoXA mmoXD	ACCAAGGARCARTTCAAG	790 bp	(2)
		CCGATCCAGATDCCRCCCCA		
	mmoX206F mmoX866R	ATCGCBAARGAATAYGCSCG	719 bp	(22)
		ACCCANGGCTCGACYTTGAA		
Sequencing	T7	TAATACGACTCACTATAGGG	variable	Invitrogen
Sequencing	T3	ATTAACCCTCACTAAAGGGA	variable	Invitrogen

**Table 2:** Total eubacterial community composition in 0.6-TCM soil on the basis of a 16S rRNA gene clone library analysis.

Phylum	Class	Order	Family	Genus	Clone library^a^	Best RDP hit^b^	% ID^c^
*Acidobacteria*	*Acidobacteria*	unspecified	unspecified	Gp3	3%	JQ712939	96
				Gp4	2%	AY094624	99
				Gp6	6%	HQ597776	99
				Gp10	6%	JQ408035	98

*Actinobacteria*	*Actinobacteria*	*Acidimicrobiales*	*Acidimicrobineae*	*Aciditerrimonas*	7%	LN573416	99

		*Actinomycetales*	*Intrasporangiaceae*	*Phycicoccus*	8%	HQ132449	99
			*Micrococcaceae*	*Arthrobacter*	5%	KF923442	99
			*Streptosporangiaceae*	*Microbispora*	1%	KF886293	96

		*Gaiellales*	*Gaiellaceae*	*Gaiella*	2%	KC554071	99

		unclassified	unclassified	unclassified	1%	FJ478842	99

*Armatimonadetes*	*Chthonomonadetes*	*Chthonomonadales*	*Chthonomonadaceae*	*Chthonomonas*	2%	GU454980	95

*Bacteroidetes*	*Sphingobacteria*	*Sphingobacteriales*	*Chitinophagaceae*	unclassified	5%	GQ487995	99

*Chloroflexi*	*Thermomicrobia*	*Sphaerobacterales*	*Sphaerobacteraceae*	*Sphaerobacter*	3%	KC432559	95

*Firmicutes*	*Bacilli*	*Bacillales*	*Bacillaceae*	*Bacillus*	16%	EU221338	99

*Proteobacteria*	*Alphaproteobacteria*	*Rhodospirillales*	*Rhodospirillaceae*	*Dongia*	3%	FJ478641	97
				*Skermanella*	1%	KF010774	99
		*Sphingomonadales*	*Sphingomonadaceae*	*Sphingomonas*	6%	JN178270	99

	*Betaproteobacteria*	*Burkholderiales*	*Alcaligenaceae*	*Derxia*	1%	GQ009540	99
			*Comamonadaceae*	unclassified	2%	EF516785	99

		*Methylophilales*	*Methylophilaceae*	*Methylobacillus*	2%	FJ444763	99

	*Gammaproteobacteria*	*Methylococcales*	*Methylococcaceae*	*Methylobacter*	7%	AY921679	98
				*Methylocaldum*	3%	HM362553	94

unclassified	Unclassified	unclassified	unclassified	unclassified	4%	JQ088371	87

a The amount of clones representing each bacterial phylotype within the clone library out of 100 screened clones.

b The best hit resulting from a comparison of each 16S rRNA gene partial sequence with sequences in the small-subunit rRNA database of the Ribosomal Database Project (RDP).

c The % of nucleotide identity revealed by a BLAST analysis of the RDP best hit.

**Table 3:** Analysis of representative *pmoA* clones obtained from the amplification of a 0.6-TCM soil extract with two primer sets (A189f/mb661r and A189f/A682r).

Group	Clone	Library with A189f/mb661r^a^	Library with A189f/A682r^a^	Best Blast hit	% ID	T-RFLP expected	T-RFLP observed
A	MD-51	1%	—	Uncultured methanotrophic bacterium clone 16 (*pmoA*)	100%	M244, H45	M242, H45
	MD-92	1%	—	Uncultured bacterium clone ZW200 (*pmoA*)	98%		

B	MD-53b	—	6%	Uncultured bacterium clone CH_118 (*pmoA*)	93%	M279, H225	M279, H226
	MD-58b	—	4%	Uncultured bacterium clone CH_118 (*pmoA*)	93%		

C	MD-2	6%	—	Uncultured bacterium clone JXL48 (*pmoA*)	94%	M208, H225	M208, H226
	MD-5	32%	—	Uncultured bacterium clone JXL48 (*pmoA*)	94%		
	MD-32	10%	—	Uncultured bacterium clone JXL48 (*pmoA*)	95%		
	MD-42	1%	—	Uncultured bacterium clone JXL48 (*pmoA*)	94%		
	MD-8b	—	15%	Uncultured bacterium clone JXL48 (*pmoA*)	94%		
	MD-105b	—	8%	Uncultured bacterium clone JXL48 (*pmoA*)	95%		

D	MD-1	17%	—	Uncultured methanotroph pmoA-61 (*pmoA*)	91%	M33, (H19)^b^	M35, (H19)^b^, H82^c^
	MD-9	1%	—	Uncultured bacterium RB18 (*pmoA*)	90%		
	MD-103b	—	57%	Uncultured methanotroph pmoA-61 (*pmoA*)	91%		

E	MD-14	10%	—	Uncultured *Methylococcus* sp. clone Xh_pmoA_CA51 (*pmoA*)	93%	M36, (H19)^b^	M37, (H19)^b^, H82^c^
	MD-57b	—	5%	Uncultured *Methylococcus* sp. clone Xh_pmoA_CA51 (*pmoA*)	93%		
	MD-5b	—	3%	Uncultured *Methylococcus* sp. clone Xh_pmoA_CA51 (*pmoA*)	93%		

F	MD-47	1%	—	Uncultured methanotrophic bacterium clone 37 (*pmoA*)	94%	M79, H85	M76, H82

G	MD-3	17%	—	Uncultured bacterium clone JXL16 (*pmoA*)	99%	M79, H100	M76, H99

H	MD-11	2%	—	Uncultured *Methylomonas* sp. clone 13RH2Omb16 (*pmoA*)	99%	(M505)^b^, H348	H350

-	MD-41	—	1%	Uncultured bacterium clone AOB-A0-21 (*amoA*)	97%	M46, H45	H45

a The % of clones representing each clone group within the 100 clones screened.

b Expected T-RFLP peaks outside of the valid T-RF range are between brackets (on the basis of the DNA fragment length standard [Rox 500]).

c Second cut.
