# Supplementary material for: Diversity of Methane-Oxidizing Bacteria in Soils from “Hot Lands of Medolla” (Italy) Featured by Anomalous High-Temperatures and Biogenic CO2 Emission
Source: Microbes Environ. 2016 Sep 17;31(4):369–77. doi: 10.1264/jsme2.ME16087 (PMC5158108; doi:10.1264/jsme2.ME16087)
Supplement: Supplementary file 1 [file 31_369_s1.pdf]

Table S1 Sequence identity (%) values among the pmoA and amoA nucleotide sequences detected in this study.

	**MD-41**	**MD-51**	**MD-92**	**MD-11**	**MD-53b**	**MD-58b**	**MD-105b**	**MD-32**	**MD-2**	**MD-8b**	**MD-42**	**MD-5**	**MD-3**	**MD-9**	**MD-1**	**MD-103b**	**MD-47**	**MD-57b**	**MD-5b**	**MD-14**
**MD-41**	100%	61%	61%	57%	59%	60%	59%	58%	58%	58%	58%	58%	63%	67%	66%	66%	65%	64%	63%	63%
**MD-51**	61%	100%	94%	63%	66%	66%	67%	67%	67%	66%	66%	67%	69%	68%	69%	68%	70%	69%	68%	69%
**MD-92**	61%	94%	100%	62%	66%	66%	67%	68%	67%	66%	67%	67%	69%	68%	67%	67%	69%	66%	66%	67%
**MD-11**	57%	63%	62%	100%	67%	67%	67%	67%	66%	67%	66%	67%	68%	70%	70%	70%	70%	71%	70%	70%
**MD-53b**	59%	66%	66%	67%	100%	99%	87%	86%	86%	86%	86%	86%	71%	72%	71%	72%	71%	72%	73%	72%
**MD-58b**	60%	66%	66%	67%	99%	100%	87%	86%	86%	86%	85%	86%	71%	72%	72%	72%	72%	73%	73%	73%
**MD-105b**	59%	67%	67%	67%	87%	87%	100%	99%	97%	97%	96%	96%	71%	73%	72%	72%	73%	72%	72%	72%
**MD-32**	58%	67%	68%	67%	86%	86%	99%	100%	98%	97%	97%	97%	72%	73%	72%	72%	74%	72%	72%	72%
**MD-2**	58%	67%	67%	66%	86%	86%	97%	98%	100%	98%	98%	98%	71%	73%	72%	72%	74%	71%	71%	72%
**MD-8b**	58%	66%	66%	67%	86%	86%	97%	97%	98%	100%	99%	99%	71%	73%	72%	72%	73%	72%	72%	71%
**MD-42**	58%	66%	67%	66%	86%	85%	96%	97%	98%	99%	100%	99%	72%	73%	72%	72%	74%	72%	72%	72%
**MD-5**	58%	67%	67%	67%	86%	86%	96%	97%	98%	99%	99%	100%	72%	73%	72%	72%	74%	72%	72%	72%
**MD-3**	63%	69%	69%	68%	71%	71%	71%	72%	71%	71%	72%	72%	100%	84%	83%	82%	87%	83%	83%	84%
**MD-9**	67%	68%	68%	70%	72%	72%	73%	73%	73%	73%	73%	73%	84%	100%	95%	95%	88%	87%	87%	88%
**MD-1**	66%	69%	67%	70%	71%	72%	72%	72%	72%	72%	72%	72%	83%	95%	100%	100%	89%	88%	88%	88%
**MD-103b**	66%	68%	67%	70%	72%	72%	72%	72%	72%	72%	72%	72%	82%	95%	100%	100%	88%	88%	88%	88%
**MD-47**	65%	70%	69%	70%	71%	72%	73%	74%	74%	73%	74%	74%	87%	88%	89%	88%	100%	89%	89%	90%
**MD-57b**	64%	69%	66%	71%	72%	73%	72%	72%	71%	72%	72%	72%	83%	87%	88%	88%	89%	100%	100%	99%
**MD-5b**	63%	68%	66%	70%	73%	73%	72%	72%	71%	72%	72%	72%	83%	87%	88%	88%	89%	100%	100%	99%
**MD-14**	63%	69%	67%	70%	72%	73%	72%	72%	72%	71%	72%	72%	84%	88%	88%	88%	90%	99%	99%	100%

Table S2 The matrix of similarities showing the Jaccard's coefficients calculated on the basis of T-RFLP profiles of the three soils collected at 0.6-, 0.7- and 2.5-m ground depths.

	0.6-TCM	0.7-TCM	2.5-TCM
0.6-TCM	1.000		
0.7-TCM	0.905	1.000	
2.5-TCM	0.714	0.619	1.000
